# Identification and profiling of circulating antigens by screening with the sera from schistosomiasis japonica patients

**DOI:** 10.1186/1756-3305-5-115

**Published:** 2012-06-11

**Authors:** Yan Lu, Bin Xu, Chuan Ju, Xiaojin Mo, Shenbo Chen, Zheng Feng, Xiaoning Wang, Wei Hu

**Affiliations:** 1 School of Biotechnology, East China University of Science and Technology, Shanghai 200237, P.R. China; 2 National Institute of Parasitic Diseases, Chinese Center for Disease Control and Prevention, Key Laboratory of Parasite and Vector biology, MOH; WHO Collaborating Center of Malaria, Schistosomiasis and Filariasis, Shanghai 200025, P.R. China; 3 Department of Microbiology and Microbial Engineering, School of Life Science, Fudan University, Shanghai 200433, P.R. China

**Keywords:** *Schistosoma japonicum*, Circulating antigens, Identification, IgY

## Abstract

**Background:**

Schistosomiasis is a chronic disease caused by trematode flatworms of the genus *Schistosoma*. The disease remains a serious public health problem in endemic countries and affects at least 207 million people worldwide. A definite diagnosis of the disease plays a key role in the control of schistosomiasis. The detection of schistosome circulating antigens (CAs) is an effective approach to discriminate between previous exposure and current infection. Different methods have been investigated for detecting the CAs. However, the components of the schistosome CAs remain unclear. In this study, we analyzed the CAs in sera of patients infected with *Schistosoma japonicum*.

**Methods:**

The parasites were collected from the infected rabbits for preparing the adult worm antigen (AWA). The hyline hens were immunized subcutaneously with AWA to produce anti-AWA IgY. The IgY was purified by water-dilution and ammonium sulfate precipitation method and identified by ELISA and Western blotting. After purification and characterization, IgY was immobilized onto the resin as a capture antibody. The circulating antigens were immune-precipitated from patients′ serum samples by direct immunoprecipitation. The precipitated proteins were separated by one-dimensional electrophoresis and analyzed by LC-MS/MS.

**Results:**

Firstly, the IgY against AWA was produced from the eggs of immunized hens by AWA, which gave a titer of 1:12800. The purified IgY was used as the capture antibody to enrich the CAs in sera of *S. japonicum* infected patients through immunoprecipitation. The CAs were determined by LC-MS/MS. There were four proteins, including protein BUD31 homolog, ribonuclease, SJCHGC06971 protein and SJCHGC04754 protein, which were identified among the CAs.

**Conclusions:**

We developed a novel method based on IgY for identification and profiling CAs in sera of *S. japonicum* infected patients. Four new CAs were identified and have potential value for further development of an antigen assay.

## Background

Schistosomiasis, also known as Bilharziasis, is a parasitic disease caused by trematode flatworms of the genus *Schistosoma*. Larval forms of the parasite, which are released by freshwater snails, penetrate the skin of the definitive hosts, human or other mammals when contacting the infested water [1-3]. Approximately 207 million people were estimated to be infected with schistosomiasis, and close to 800 million people were at risk of infection [4]. Schistosomiasis causes significant morbidity and mortality in developing countries. A meta-analysis assigned 2-15% disability weight associated with chronic schistosome infection [5].

Sensitive diagnoses, monitoring of the disease transmission and evaluation of chemotherapeutic interventions, are of primary importance for the improvement of control and prevention strategies for schistosomiasis [6]. Schistosomiasis can be diagnosed by direct or indirect methods: a) direct parasitological examinations to detect parasite eggs in fecal/urine samples or in the tissues; b) direct approaches to detect the schistosome-derived antigens in the circulation and excreta; c) indirect immunological tests to detect the specific antibodies induced against the different stages of the parasite in blood [7,8]. Direct parasitological diagnosis techniques are labor-intensive and time-consuming. Moreover, their low sensitivities would result in under-estimation in prevalence and infection intensity, particularly in the areas with low prevalence or after intervention [9,10]. Immunological diagnoses are applied most widely to detect the antibodies due to a higher sensitivity. However, antibody-based serological assays do not discriminate between active and past infections, and thus could not be used to evaluate therapeutic efficacy since specific antibodies continue to be present for a long time after the worms have disappeared [10,11].

Therefore, detection of circulating antigens has been used for the diagnosis of schistosomiasis because these antigens could be demonstrated in the circulation and excreta of infected individuals and the antigen levels have been found to correlate well with parasitic load [12,13]. This method has proved to be an effective way to assess the active infections and the effects of treatments in endemic areas with high sensitivity and specificity [7,14,15]. Furthermore, detection of these antigens has provided a valuable tool for population screening, and to study the sero-epidemiology of the disease [16,17]. A test has been developed to detect circulating cathodic antigen (CCA) in urine for the diagnosis of *Schistosoma mansoni* as a rapid diagnostic test in cassette form. Although the assay shows similar sensitivity to the Kato-Katz method for *S. mansoni* diagnosis, it is still an attractive tool due to its fast and easy application for the large-scale screening in control programs [18,19]. Moreover, a sandwich time-resolved fluoroimmunoassay (TRFIA) for detecting the circulating antigen 14-3-3 of *S. japonicum* in rabbits could reach higher positive rates compared to ELISA within the first 21 days post-infection. It is demonstrated to be a good early diagnostic method for active schistosome infection [20].

According to the different developmental stages of the schistosome, the circulating antigens can be classified into cercarial antigens, adult worm associated antigens (e.g. tegument or gut-associated), and egg antigens [7].The major circulating antigens belong to the group of the adult worm gut-associated circulating antigens. These antigens are released into the circulation of the host at regular time intervals from the gut of adult schistosomes [7,21]. So far, most research has focused on the circulating anodic antigen (CAA) and the circulating cathodic antigen (CCA) [22-27]. In addition to CAA and CCA, few of the other circulating antigens have been characterized.

We intend to characterize more circulating antigens by a new method based on egg yolk immunoglobulin (IgY). The IgY has been recognized as an alternative source of polyclonal antibodies. The use of chicken IgY instead of mammalian antibodies brings great benefit concerning the welfare of the immunized animals, due to non-invasive antibody harvesting with the added convenience of simple egg collection. An additional advantage is the fast and simple IgY isolation from egg yolk [28]. In addition, IgY do not activate the mammalian complement system [29], or bind to rheumatoid factors (RF) [30], or show interaction with human and bacterial Fc receptors [31,32]. Because of these advantages, IgY has been used for diagnosis in different diseases [33-35]. Recently, a novel immunomagnetic bead ELISA using IgY against SEA as a capture antibody (IgY-IMB-ELISA) was applied to detect CAs in sera of murine schistosomiasis and the serum samples of persons with schistosomiasis. This method appeared to be sensitive and specific by using 100μl serum samples for diagnosis of schistosome infection and also valuable in judging the efficacy of chemotherapy in schistosomiasis [36,37].

In the present study, we used IgY as the capture antibody to concentrate the circulating antigens in the sera of schistosomiasis japonica patients through immunoprecipitation. Then the antigens were identified by liquid chromatography-tandem mass spectrometry (LC-MS/MS). This was the first study for profiling CAs of *S. japonicum*, the findings of which might be of informative significance for development of new diagnostic agents of schistosomiasis japonica.

## Methods

### Schistosome materials

Each laboratory rabbit (*Oryctolagus cuniculus*) was percutaneously infected with 1000 *S. japonicum* cercariae isolated from the infected *Oncomelania hupensis* snails in the field. After challenge infection, the adult worms were collected by perfusing the hepatic portal system and mesenteric veins of the rabbits at 42 days post infection. The worms were washed at least three times with normal saline to remove the host tissues [38].

### Antigen preparation

Briefly, adult S. *japonicum* worms (Chinese strain) were suspended in the buffer (10 mM KCl, 10 mM Tris-Cl pH7.5, 1 mM EDTA, 10nM β-mercaptoethanol, 5 mM DTT, 20% glycerol), homogenized with a tissue grinder, frozen and thawed three times, and then sonicated with three cycles at 100 Hz for 60 seconds each [39,40].

The antigen solution was a homogenate including the total soluble proteins and insoluble proteins of adult worm. The concentration of the suspension was determined by Bradford protein assay kit (TIANGEN, China) according to the manufacturer′s instructions. The prepared adult worm antigen (AWA) obtained was aliquoted and stored at −20°C until use.

### Preparation and characterization of IgY

AWA was formulated with 2 volumes of either Freund complete (prime) or Freund incomplete (two boost) adjuvant. 28-week-old hyline hens were immunized subcutaneously with AWA four times at an interval of 14 days with a dose of 0.5 ml (1.8 mg protein), while the AWA in PBS was used for the last immunization. The hens were maintained in a standard SPF (specific pathogen-free) condition. Chicken eggs were collected daily before immunization and 7 days after the last immunization. The eggs from unimmunized chicken were also collected as a normal control. The IgY antibody was purified from egg yolk by water-dilution and ammonium sulfate precipitation method. The egg white and egg yolk membrane were removed after breaking the eggs; the egg yolk was diluted with 9 volumes of distilled water, and mixed by stirring fully. The pH value of the solution was adjusted to 5.1-5.4 with HCl and stored at 4°C over night. The supernatant was filtered through the filter papers, and then centrifuged at 10000 rpm for 10 min at 4°C. The crude extraction suspension was mixed with 50% (V/V) saturated ammonium sulfate solution and stirred at 4°C for 2 h. After centrifugation, the precipitate was collected and dissolved in 0.01 M phosphate buffered saline (PBS, pH 7.4). The solution was re-precipitated by adding 33% (V/V) saturated ammonium sulfate. The precipitate was dissolved in PBS in an equal volume to the original egg yolk volume and dialyzed against distilled water, and then PBS to remove the NH^4+^[41-43].

Protein content of purified IgY was checked by Bradford protein assay kit (TIANGEN, China). The same amount of IgY proteins before and after purification were analyzed by SDS-PAGE. The gels were stained by Coomassie brilliant blue to observe the protein bands.

The antibody titer was estimated by indirect ELISA. The Nunc-Immuno plates (MaxiSorp F96, Thermo, USA) were coated with 0.125 μg AWA per well in 0.06 M carbonate coating buffer (pH 9.6) and stored overnight at 4°C. After washing three times with PBS containing 0.1% Tween 20, the plates were blocked with 1% BSA diluted in PBS overnight at 4°C. The purified IgY samples were diluted to 1:400, 1:800, etc. until 1:20480, then, 100μl was dispensed per well in duplicate onto the plates. The IgY from unimmunized egg yolk was used as negative control. The plates were incubated at 37°C for 2 h. The peroxidase-conjugated rabbit anti-chicken secondary antibody (Sigma, USA) was used at a 1:20000 dilution and the plates were incubated at 37°C for 1 h. The substrate, 3, 3′, 5, 5′-tetramethylbenzidine (TMB, TIANGEN, China) was used for the assay. The optical density (OD) was measured at 450 nm on a microplate reader (Model 680 XR, Bio-Rad) [42].

Western blotting was employed to evaluate the immunoreactivity of IgY. Briefly, the equal amounts of AWA were separated in 10% SDS-PAGE and then transferred onto a 0.45 μm nitrocellulose membrane (Amersham Biosciences-GE Healthcare, USA) at 0.22 A for 1.5 h. After blocking with 3% BSA diluted in PBS overnight at room temperature (RT), the membrane was cut as vertical strips of 4 mm wide and individually treated for 2 h at RT with IgY from immunized or unimmunized egg yolk at a dilution of 1:400. The strips were washed at least three times in PBS containing 0.1% Tween 20 then incubated with peroxidase-conjugated rabbit anti-chicken secondary antibody (Sigma, USA) diluted 1:2000 for 1 h at RT. After washing, the substrate 3, 3′-Diaminobenzidine (DAB, Sigma, USA) was added to develop the color reaction [44,45].

### Direct immunoprecipitation

Ten serum samples of schistosomiasis patients and normal human sera were randomly selected from a sera bank of National Institute of Parasitic Diseases (NIPD), China CDC. The serum samples of patients were collected from the endemic areas in Jiangxi provinces, P.R. China. The diagnosis was made by the Kato-Katz method for schistosome eggs in the feces. Nine slides were prepared from three consecutive stool samples and each slide was examined blind by two trained technicians. The results were recorded as eggs per gram feces (EPG) and the EPG of ten patients were among 11–283. The patients included two women and eight men, with ages ranging from 12 to 59 (Additional file 1: Table S1). The normal sera were collected from healthy people living in Shanghai who had never traveled to schistosomiasis endemic areas.

The CAs were enriched using sera of schistosomiasis patients according to the protocol of Pierce Direct IP Kit (Thermo, USA), and the sera from healthy individuals were used as a negative control. The enrichment was carried out by incubation of 100 μl of the AminoLink Plus Coupling Resin slurry with 50 μg purified antibody at RT for 120 minutes. The pure IgY from immunized egg yolk was immobilized onto the aldehyde-activated beaded agarose resin. 1 ml of pooled positive sera (10 individual serum samples were pooled) was added to the antibody-coupled resin in a spin column, the column was incubated with gentle shaking for 1 h at 4°C, to form the antibody-antigen complex. The complex was washed five times with tris-buffered saline (TBS) to remove non-bound material and then eluted with 50μl elution buffer (pH 2.8) to dissociate the bound antigen from the antibody. The target proteins were analyzed by 12% SDS-PAGE. Meanwhile, a control immunoprecipitation was done by using the IgY from unimmunized egg yolk. A freeze drying method was applied to concentrate the proteins before mass spectrometry.

### Mass spectrometry analyses of the antigens

The protein mixtures were denatured in the loading buffer for SDS-PAGE and separated by one-dimensional electrophoresis. The target lane of the gel was cut equally into ten slices; the size of a slice was about 5 × 5 mm and numbered 1–10 from the top to the bottom. Each slice was minced into 1 × 1 mm size pieces and subsequently subjected to in-gel digestion with modified trypsin (Roche) according to standard procedures. Peptides were extracted by sonication with 50% ACN, 5%FA in ddH2O. Finally, the extracted tryptic digests were concentrated in a speedvac to a final volume of ~10μl prior to mass spectrometric analysis. After pre-preparation, the peptide mixtures from the gel slices were analyzed by LC-MS/MS as previously described [38,46,47] . In brief, the tryptic digests were then loaded onto a reverse phase (RP) trap column (C18, 5 μm, 300 Å, 300 mm id × 5 mm, Waters) for enrichment at a flow rate of 10 μl/min. The trap column was sequentially connected in-line with an analytical 75 μm × 150 mm C18 column (Waters) and the peptide mixtures were eluted into SYNAPT G2 (Waters) at a flow rate of 200 nl/min. NanoUPLC (Waters) was used to deliver mobile phases A (0.5% acetic acid in water) and B (0.5% acetic acid in ACN) at a linear gradient from 5% B to 50% B within 60 min, along with a gradient from 50% B to 90% B within 30 min and then 90% B for 15 min. A spray voltage of 3200 V was applied to a 10 μm id PicoTip nanospray emitter (New Objective) connected at the end of the analytical column through a stainless union joint (Valco Instrument) to give a steady spray.

The data were post acquisition lock mass corrected using the doubly charged monoisotopic ion of [Glu1]-fibrinopeptide B. The reference sprayer was sampled with a frequency of 30 s. Accurate mass LC-MS data were collected in an alternating, low energy, and elevated-energy mode of acquisition. The spectral acquisition time in each mode was 0.9 s. In low energy MS mode, data were collected at constant collision energy of 4 eV. In elevated-energy MS mode, the collision energy was ramped from 15 to 55 eV during each 0.9 s integration. One cycle of low and elevated-energy data was acquired every 1.84 s.The scan window was set from m/z 100 to 1800.

The LC-MS/MS spectra were searched against the *S. japonicum* protein databases using MASCOT software (hettp://http://www.matrixscience.com, Matrix Science) as previously described [38,46]. To determine whether these peptides originated from the schistosome or the host, the MSE DATA were searched against the human protein databases (IPI, HUMAN, V3.72) using PLGS 2.4 (Waters). Searching parameters as follows: the Value of Min Fragment Ion Matches per Peptide was 3, the Value of Min Fragment Ion Matches per Protein was 7, and the Value of Min Peptide Matches per Protein 1; Trypsin was set as digest reagent, the allowed number of Missed Cleavages was 2; Carbamidomethyl C was set as fixed modification, Oxidation M and Phosphoryl STY were set as variable modifications. The False Positive Rate was less than 1%. The peptides identified were also compared with the protein sequences of schistosome and human by using the BLAST program.

## Results

### Preparation of the worm antigen

The parasites were collected from the infected rabbits, and used to prepare the adult worm antigen (AWA). Finally, the homogenate antigen solution, which contains the total soluble proteins and insoluble proteins of adult worm, were obtained. The concentration of the crude solution was 10.8 mg/ml by Bradford protein assay.

### Analysis of IgY

We used the water dilution method to obtain a supernatant with crude egg yolk antibody (water-soluble fraction, WSF), and then the IgY was precipitated by ammonium sulfate with better purity. The concentration of purified IgY was 7.44 mg/ml. From each immunized egg yolk, about 75 mg of IgY was extracted. IgY is a big biological molecule with a molecular weight of 180 kDa and consists of two larger subunits and two smaller subunits. The molecular weights of the larger subunits and smaller subunits were about 66 kDa and 30 kDa respectively. Under reducing conditions, the disulfide bond could be broken down, resulting in the separation of larger subunits and smaller subunits. SDS-PAGE analysis showed two protein bands, the heavy chain was 62 kDa and the light chain was 37 kDa.

**Figure 1:**
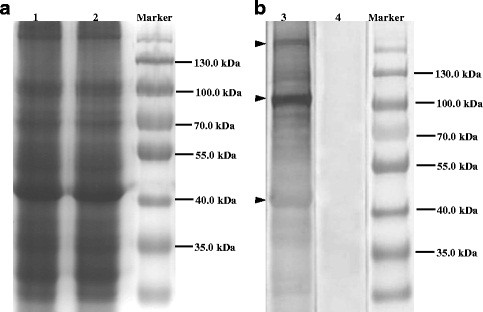
**Immunoprecipitation of the circulating antigens.** By using the anti-AWA IgY, several protein bands with different molecular weights could be immunoprecipitated from the patients′ sera, while only nonspecific protein bands could be immunoprecipitated from healthy peoples′ sera. By using IgY from unimmunized egg yolk, the same nonspecific bands were observed both in patients′ sera and healthy peoples′ sera. Marker: protein size standard, 1: IgY, 2: Flow-through after antibody coupling, 3: Flow-through after immunoprecipitation from patients′ sera, 4: Flow-through after immunoprecipitation from healthy peoples′ sera, 5: Eluate from patients′ sera, 6: Eluate from healthy peoples′ sera. A: Immunoprecipitation of the circulating antigens by using IgY from immunized egg yolk.

After immunization and purification, the titer of IgY was 1:12800. Equivalent amounts of AWA were loaded onto the different lanes of the gels. One gel was used for a Western blotting assay and the other gel was stained by Coomassie brilliant blue as a control. The results of Western blotting showed that different protein bands in AWA could be recognized by the IgY from immunized egg yolk and no specific band was recognized by the IgY from the unimmunized egg yolk (Figure 1).

### Direct immunoprecipitation

**Figure 2:**
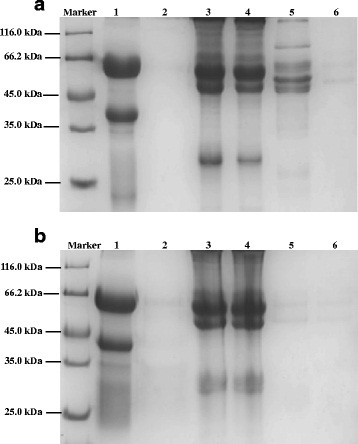
**Immunoprecipitation of the circulating antigens.** The circulating antigens were precipitated by the anti-AWA IgY. SDS-PAGE was used to analyze the immunoprecipitated proteins.

The circulating antigens were immune-precipitated from serum samples by using the Thermo Scientific Pierce Direct IP Kit. Firstly, IgY was immobilized on the beads, the sera were incubated with antibody-binding resin on the spin column. The antigens were eluted from the beaded agarose into the elution buffer by means of microcentrifuge spin cups. By using the anti-AWA IgY, several protein bands with different molecular weights could be immunoprecipitated from the patients′ sera, while only nonspecific protein bands could be immunoprecipitated from healthy peoples′ sera. By using IgY from unimmunized egg yolk, the same nonspecific bands were observed both in patients′ sera and healthy persons′ sera (Figure 2). The proteins precipitated from patients′ sera by the anti-AWA IgY were expected as circulating antigens. The proteins were concentrated with the final concentration of 0.27 mg/mL for the following MS analysis.

Marker: protein size standard, 1: IgY, 2: Flow-through after antibody coupling, 3: Flow-through after immunoprecipitation from patients′ sera, 4: Flow-through after immunoprecipitation from healthy peoples′ sera, 5: Eluate from patients′ sera, 6: Eluate from healthy peoples′ sera. A: Immunoprecipitation of the circulating antigens by using IgY from immunized egg yolk. B: Immunoprecipitation of the circulating antigens by using IgY from unimmunized egg yolk

### Identification of the circulating antigens by mass spectrometry

*S. japonicum* circulating antigens were analyzed by LC-MS/MS followed by forward and reverse database searching and BLAST program. We characterized four *S. japonicum* proteins from the gel bands (Table 1, Additional file 2: Table S2) as *S. japonicum* circulating antigens, including protein BUD31 homolog (CAX70049.1), ribonuclease (CAX70531.1), SJCHGC06971 protein (AAX28592.2) and SJCHGC04754 protein (AAX28446.2). The molecular weights of four proteins ranged from 10 to 22 kDa. The SJCHGC06971 protein and SJCHGC04754 protein were identified from the eighth slice, protein BUD31 homolog was identified from the ninth slice and the ribonuclease was identified from the tenth slice.

**Table 1:** **Proteins in sera of*S. japonicum*patients identified by LC-MS/MS**

**Accession no.**	**Annotation**	**mw(Da)**	**pI**	**PLGS score**	**Peptides**	**Coverage**
CAX70049.1	Protein BUD31 homolog	16962	8.9205	733.3332	9	30.5556
CAX70531.1	ribonuclease	10766	6.0502	579.0666	2	61.2245
AAX28592.2	SJCHGC06971 protein	20168	8.0665	419.5011	5	43.956
AAX28446.2	SJCHGC04754 protein	21607	5.2317	290.0222	2	7.3298

## Discussion

Advanced diagnosis for the disease plays a crucial role in the control of schistosomiasis, especially when the transmission levels are not very high. Detection of circulating antigens is one of the most accurate diagnostic methods for distinguishing between the active or past infection and for evaluation of the chemotherapy efficiency.

Apart from the CAA and CCA, little else is known about schistosome antigens in the circulation. In this study, we established a new method for profiling the CAs of *S. japonicum* by direct immunoprecipitation based on IgY and mass spectrometry. It is the first report on profiling CAs which schistosomes have excreted into the sera. Compared to the mammalian antibodies, IgY had a higher specificity, sensitivity and stability [29-32]. The IgY against AWA was used as the capture antibody to enrich the CAs through immunoprecipitation in our research. Immunoprecipitation could be used to isolate and concentrate a particular protein from a sample containing thousands of different proteins. In contrast to the traditional methods, the Direct IP Kit used an amine-reactive support that does not contain Protein A or Protein G and did not need a cross linker for covalent immobilization. Furthermore, this method could couple any primary amine-containing molecule, unlike the other methods requiring certain species and subclass of the antibody that binds strongly to Protein A or Protein G. The antibody was irreversibly attached to the agarose beads so that co-elution of heavy and light chains with the purified protein is minimized. Only antigens were eluted by the procedure, enabling them to be identified and further analyzed without interference from antibody fragments.

Moreover, Deelder’s group analyzed the composition of CAA and CCA years ago. The results indicated that CCA is O-glycosylated mostly via GalNAc-Thr and CAA is a glycoprotein, O-glycosylated at Thr. The major carbohydrate fraction of CCA comprised a population of polysaccharides, containing Lewis x repeating units (−3) Gal beta (1–4) [Fuc alpha (1–3)] GlcNAc beta (1-) [48]. While the major carbohydrate chains of CAA had a novel polysaccharide structure, consisting of a branched disaccharide repeating unit containing 2-acetamido-2-deoxy-beta-D- galactopyranose (beta-D-Galp-NAc) and beta-D-glucopyranuronic acid (beta-D-GlcpA) [49]. However, we analyzed the glycosylation sites for these four proteins by online tools (https://www.predictprotein.org/http://www.cbs.dtu.dk/services/) and found no O-glycosylation sites among them (Data not shown here). The result implied that the four proteins may be new CAs except CAA and CCA.

Among the four proteins, protein BUD31 homolog shared 99% (143/144) identity with g10 protein homolog of *S. mansoni*. Protein BUD31 and G10 protein were members of G10 superfamily. The G10 family was involved in transcription/cell division and chromosome partitioning. The conserved domain BUD 31 was related to cell cycle control. Protein BUD 31 had been found in multiple splicing related protein complexes [50,51].

The ribonuclease of *S. japonicum* (CAX70531.1) showed 98% (96/98) identity with *S. mansoni* ribonuclease (XP002581286.1). However, the alignment of this ribonuclease (CAX70531.1) revealed a very low identity (<15%) with the omega-1 (ABB73002.1, *S. mansoni*), which had previously been identified as a hepatotoxic ribonuclease [52].Omega-1was a 31 kDa monomeric glycoprotein with an isoelectric point (pI) of greater than 9 released from *S. mansoni* eggs [52,53]. The result of ELISA with sera from mice and humans infected with different schistosome species showed that omega-1 was specific to *S. mansoni*[52]. Omega-1 was capable of conditioning human monocyte-derived dendritic cells (DCs) *in vitro* to drive T helper 2 (Th2) polarizations with similar characteristics as whole SEA [54]. Omega-1 also affected the adherence properties and morphology of DCs and omega-1-exposed DCs displayed pronounced cytoskeletal changes and exhibited decreased antigen-dependent conjugate formation with CD4+ T cells [55]. Since the ribonuclease in this paper is a 98-amino acid protein with a molecular weight of 11 kDa and a pI of 6, the protein might represent a new ribonuclease of schistosomes.

The SJCHGC06971 protein shared 65% identity with *S. mansoni* centaurin/arf-related. The SJCHGC04754 protein belonged to the AAT-I Superfamily and showed 55% identity with *Ascaris suum* aromatic L-amino-acid decarboxylase (AADC). AADC, which was responsible for the biosynthesis of serotonin in mammalian systems, was demonstrated in isolated muscle and intestinal tissue of adult female *A. suum*[56].

Although there are some previous reports about Protein BUD31 and ribonuclease, the potential values for the diagnosis are still being defined. SJCHGC06971 protein and SJCHGC04754 protein had no annotation and had not been characterized in the adult worm of schistosomes. Furthermore, the four proteins in this paper or the *S. mansoni* homologues had not been reported from the proteomic analysis of *S.mansoni* egg secretions, the schistosome tegumental proteins, adult *S. mansoni* gut contents and excretory/secretory proteins of adult *S. japonicum* worms [38,57-60]. More work is needed for the further study of these proteins.

Identification of all the CAs components was important for understanding how schistosomes interact with the host. It is believed that the information provided in this study may facilitate the development of new diagnostic antigens for schistosomiasis control.

Detection of CAs secreted by living parasites was a desirable way to differentiate between active and past infections; however, suitable circulating antigens for this purpose remained unavailable. On the other hand, the natural antigens were difficult to isolate in large amounts. The development of genetic-engineering techniques has allowed the production of recombinant antigens in sufficient quantities for large-scale testing. Pure or single-molecule antigen might also improve the specificity of immunodiagnostic tests. Future studies will aim at finding high-abundance circulating antigens, which will be cloned and expressed. Preparation of monoclonal antibodies (mAbs) to the recombinant antigens, then the IgY and mAbs will be applied for antigen detection through the double sandwich ELISA. Such investigations should offer new perspectives on diagnosis of schistosomiasis or evaluation of the efficacy of chemotherapy.

## Conclusions

Circulating antigens could be used for distinguishing the active or past schistosome infection, as well as for evaluation of the chemotherapy efficacy. In this study, we developed a novel method for identification and profiling CAs in sera of *S. japonicum* infected patients. This method based on IgY, direct immunoprecipitation and LC-MS/MS. Four new CAs, which have potential value for further development of an antigen assay, were identified. It was the first report on profiling CAs of *S. japonicum* and the results were helpful to find new diagnostic antigens for assessing active infections and evaluating the efficacy of treatments.

### Ethics statement

The study and collection of serum specimens were approved by the Ethics Committee of NIPD, China CDC (IRB00000831). All animal experimental procedures were performed according to the National Guidelines for Laboratory Animal Welfare (National Science and Technology Committee, 1988 and Ministry of Science and Technology of People′s Republic of China, 2006).

## Abbreviations

CA, Circulating antigen; AWA, Adult worm antigen; CAA, Circulating anodic antigen; CCA, Circulating cathodic antigen; RF, Rheumatoid factor; LC-MS/MS, Liquid chromatography-tandem mass spectrometry; WSF, Water-soluble fraction; SEA, Soluble egg extract; SPF, Specific pathogen-free; ELISA, Enzyme-linked immunosorbent assay; TMB, 3, 3′, 5, 5′-tetramethylbenzidine; SDS-PAGE, Sodium dodecyl sulfate-polyacrylamide gel electrophoresis; NIPD, National Institute of Parasitic Diseases; CDC, Centers for Disease Control and Prevention; EPG, Eggs per gram feces; RT, Room temperature; TBS, Tris-buffered saline.

## Competing interests

The authors declare that they have no competing interests related to this article.

## Authors′ contributions

WH, XNW, YL and FZ conceived and designed the research. YL and BX performed the experiments and analyzed the data. CJ, XJM and SBC contributed materials and helped in study implementation. YL and WH wrote and revised the manuscript. All authors read and approved the final version of the manuscript.

## Supplementary Material

Additional file 1 **Table S1.** The detailed information of schistosomiasis patients. Click here for file

Additional file 2 **Table S2.** The peptide sequences of the associated proteins. Click here for file

## References

[B1] King CH Toward the elimination of schistosomiasis N Engl J Med 2009 360 106 109 10.1056/NEJMp0808041 19129524

[B2] Hotez PJ Molyneux DH Fenwick A Kumaresan J Sachs SE Sachs JD Savioli L Control of neglected tropical diseases N Engl J Med 2007 357 1018 1027 10.1056/NEJMra064142 17804846

[B3] Hotez PJ Bethony JM Diemert DJ Pearson M Loukas A Developing vaccines to combat hookworm infection and intestinal schistosomiasis Nat Rev Microbiol 2010 8 814 826 10.1038/nrmicro2438 20948553

[B4] Steinmann P Keiser J Bos R Tanner M Utzinger J Schistosomiasis and water resources development: systematic review, meta-analysis, and estimates of people at risk Lancet Infect Dis 2006 6 411 425 10.1016/S1473-3099(06)70521-7 16790382

[B5] King CH Dickman K Tisch DJ Reassessment of the cost of chronic helmintic infection: a meta-analysis of disability-related outcomes in endemic schistosomiasis Lancet 2005 365 1561 1569 10.1016/S0140-6736(05)66457-4 15866310

[B6] Balog CI Alexandrov T Derks RJ Hensbergen PJ van Dam GJ Tukahebwa EM Kabatereine NB Thiele H Vennervald BJ Mayboroda OA Deelder AM The feasibility of MS and advanced data processing for monitoring Schistosoma mansoni infection Proteomics Clin Appl 2010 4 499 510 21137067 10.1002/prca.200900158

[B7] van Lieshout L Polderman AM Deelder AM Immunodiagnosis of schistosomiasis by determination of the circulating antigens CAA and CCA, in particular in individuals with recent or light infections Acta Trop 2000 77 69 80 10.1016/S0001-706X(00)00115-7 10996122

[B8] Zhou YB Zheng HM Jiang QW A diagnostic challenge for Schistosomiasis japonica in China: consequences on praziquantel-based morbidity control Parasit Vectors 2011 4 194 10.1186/1756-3305-4-194 21981948 PMC3195757

[B9] Midzi N Butterworth AE Mduluza T Munyati S Deelder AM van Dam GJ Use of circulating cathodic antigen strips for the diagnosis of urinary schistosomiasis Trans R Soc Trop Med Hyg 2009 103 45 51 10.1016/j.trstmh.2008.08.018 18951599

[B10] van Dam GJ Wichers JH Ferreira TM Ghati D van Amerongen A Deelder AM Diagnosis of schistosomiasis by reagent strip test for detection of circulating cathodic antigen J Clin Microbiol 2004 42 5458 5461 10.1128/JCM.42.12.5458-5461.2004 15583265 PMC535219

[B11] Legesse M Erko B Field-based evaluation of a reagent strip test for diagnosis of schistosomiasis mansoni by detecting circulating cathodic antigen (CCA) in urine in low endemic area in Ethiopia Parasite 2008 15 151 155 18642508 10.1051/parasite/2008152151

[B12] Kahama AI Kremsner PG van Dam GJ Deelder AM The dynamics of a soluble egg antigen of Schistosoma haematobium in relation to egg counts, circulating anodic and cathodic antigens and pathology markers before and after chemotherapy Trans R Soc Trop Med Hyg 1998 92 629 633 10.1016/S0035-9203(98)90789-1 10326106

[B13] Elliott DE Schistosomiasis. Pathophysiology, diagnosis, and treatment Gastroenterol Clin North Am 1996 25 599 625 10.1016/S0889-8553(05)70265-X 8863042

[B14] Legesse M Erko B Field-based evaluation of a reagent strip test for diagnosis of Schistosoma mansoni by detecting circulating cathodic antigen in urine before and after chemotherapy Trans R Soc Trop Med Hyg 2007 101 668 673 10.1016/j.trstmh.2006.11.009 17368699

[B15] de Carvalho Souza A Kuil J Maljaars CE Halkes KM Vliegenthart JF Kamerling JP Synthesis and conjugation of oligosaccharide analogues of fragments of the immunoreactive glycan part of the circulating anodic antigen of the parasite Schistosoma mansoni Org Biomol Chem 2004 2 2972 2987 10.1039/b410241j 15480463

[B16] Corstjens PL van Lieshout L Zuiderwijk M Kornelis D Tanke HJ Deelder AM van Dam GJ Up-converting phosphor technology-based lateral flow assay for detection of Schistosoma circulating anodic antigen in serum J Clin Microbiol 2008 46 171 176 10.1128/JCM.00877-07 17942645 PMC2224263

[B17] Deelder AM Qian ZL Kremsner PG Acosta L Rabello AL Enyong P Simarro PP van Etten EC Krijger FW Rotmans JP Quantitative diagnosis of Schistosoma infections by measurement of circulating antigens in serum and urine Trop Geogr Med 1994 46 233 238 7825226

[B18] Shane HL Verani JR Abudho B Montgomery SP Blackstock AJ Mwinzi PN Butler SE Karanja DM Secor WE Evaluation of urine CCA assays for detection of Schistosoma mansoni infection in Western Kenya PLoS Negl Trop Dis 2011 5 e951 10.1371/journal.pntd.0000951 21283613 PMC3026766

[B19] Coulibaly JT Knopp S N'Guessan NA Silue KD Furst T Lohourignon LK Brou JK N'Gbesso YK Vounatsou P N'Goran EK Utzinger J Accuracy of urine circulating cathodic antigen (CCA) test for Schistosoma mansoni diagnosis in different settings of Cote d'Ivoire PLoS Negl Trop Dis 2011 5 e1384 10.1371/journal.pntd.0001384 22132246 PMC3222626

[B20] Qian CY Huang B Yu CX Zhang J Yin XR Wang J Song LJ Zhang W Ke XD Detection of the circulating antigen 14-3-3 protein of Schistosoma japonicum by time-resolved fluoroimmunoassay in rabbits Parasit Vectors 2011 4 95 10.1186/1756-3305-4-95 21619661 PMC3115898

[B21] Vermeer HJ van Dam GJ Halkes KM Kamerling JP Vliegenthart JF Hokke CH Deelder AM Immunodiagnostically applicable monoclonal antibodies to the circulating anodic antigen of Schistosoma mansoni bind to small, defined oligosaccharide epitopes Parasitol Res 2003 90 330 336 10.1007/s00436-003-0860-3 12695908

[B22] Polman K De Vlas SJ Van Lieshout L Deelder AM Gryseels B Evaluation of density-dependent fecundity in human Schistosoma mansoni infections by relating egg counts to circulating antigens through Deming regression Parasitology 2001 122 161 167 11272646 10.1017/s0031182001007193

[B23] Polman K Diakhate MM Engels D Nahimana S Van Dam GJ Falcao Ferreira ST Deelder AM Gryseels B Specificity of circulating antigen detection for schistosomiasis mansoni in Senegal and Burundi Trop Med Int Health 2000 5 534 537 10.1046/j.1365-3156.2000.00600.x 10995094

[B24] Al-Sherbiny MM Osman AM Hancock K Deelder AM Tsang VC Application of immunodiagnostic assays: detection of antibodies and circulating antigens in human schistosomiasis and correlation with clinical findings AmJTrop Med Hyg 1999 60 960 966 10.4269/ajtmh.1999.60.960 10403328

[B25] De Clercq D Sacko M Vercruysse J vanden Bussche V Landoure A Diarra A Gryseels B Deelder A Circulating anodic and cathodic antigen in serum and urine of mixed Schistosoma haematobium and S. mansoni infections in Office du Niger, Mali Trop Med Int Health 1997 2 680 685 10.1046/j.1365-3156.1997.d01-354.x 9270735

[B26] Deelder AM van Dam GJ Kornelis D Fillie YE van Zeyl RJ Schistosoma: analysis of monoclonal antibodies reactive with the circulating antigens CAA and CCA Parasitology 1996 112 Pt 1 21 35 8587799 10.1017/s0031182000065045

[B27] Standley C Lwambo N Lange C Kariuki H Adriko M Stothard J Performance of circulating cathodic antigen (CCA) urine-dipsticks for rapid detection of intestinal schistosomiasis in schoolchildren from shoreline communities of Lake Victoria Parasit Vectors 2010 3 7 10.1186/1756-3305-3-7 20181101 PMC2828997

[B28] Tini M Jewell UR Camenisch G Chilov D Gassmann M Generation and application of chicken egg-yolk antibodies Comp Biochem Physiol A Mol Integr Physiol 2002 131 569 574 10.1016/S1095-6433(01)00508-6 11867282

[B29] Larsson A Wejaker PE Forsberg PO Lindahl T Chicken antibodies: a tool to avoid interference by complement activation in ELISA J Immunol Methods 1992 156 79 83 10.1016/0022-1759(92)90013-J 1431165

[B30] Larsson A Karlsson-Parra A Sjoquist J Use of chicken antibodies in enzyme immunoassays to avoid interference by rheumatoid factors Clin Chem 1991 37 411 414 2004449

[B31] Carlander D Kollberg H Wejaker PE Larsson A Peroral immunotherapy with yolk antibodies for the prevention and treatment of enteric infections Immunol Res 2000 21 1 6 10.1385/IR:21:1:1 10803878 PMC7090601

[B32] Carlander D Stalberg J Larsson A Chicken antibodies: a clinical chemistry perspective Ups J Med Sci 1999 104 179 189 10.3109/03009739909178961 10680951

[B33] Shin SJ Lee SS Manning EJ Collins MT Production of and applications for a polyclonal IgY diagnostic reagent specific for Mycobacterium avium subsp. paratuberculosis J Microbiol 2009 47 600 609 10.1007/s12275-009-0052-7 19851733

[B34] Empey Campora C Hokama Y Yabusaki K Isobe M Development of an enzyme-linked immunosorbent assay for the detection of ciguatoxin in fish tissue using chicken immunoglobulin Y J Clin Lab Anal 2008 22 239 245 10.1002/jcla.20256 18623134 PMC6649007

[B35] Juliarena M Gutierrez S Ceriani C Chicken antibodies: a useful tool for antigen capture ELISA to detect bovine leukaemia virus without cross-reaction with other mammalian antibodies Vet Res Commun 2007 31 43 51 17187239 10.1007/s11259-006-3422-1

[B36] Lei JH Liu WQ Sun CS Tang CL Li MJ Chen YL Li YL Detection of circulating antigen in serum of mice infected with Schistosoma japonicum by immunomagnetic bead ELISA based on IgY Acta Trop 2009 111 39 43 10.1016/j.actatropica.2009.02.012 19426661

[B37] Lei JH Su BT Xu H Shen JL Guan XH Feng ZQ Li YL Xu MX Liu WQ Evaluation of an IgY-based immunomagnetic enzyme-linked immunosorbent assay system for detection of circulating Schistosoma japonicum antigen in serum samples from patients in China AmJTrop Med Hyg 2011 85 1054 1059 10.4269/ajtmh.2011.11-0051 PMC3225151 22144443

[B38] Liu F Cui SJ Hu W Feng Z Wang ZQ Han ZG Excretory/secretory proteome of the adult developmental stage of human blood fluke, Schistosoma japonicum Mol Cell Proteomics 2009 8 1236 1251 10.1074/mcp.M800538-MCP200 19299421 PMC2690496

[B39] Li YS Ross AG Sleigh AC Li Y Waine GJ Williams GJ Tanner M McManus DP Antibody isotype responses, infection and re-infection for Schistosoma japonicum in a marshland area of China Acta Trop 1999 73 79 92 10.1016/S0001-706X(99)00019-4 10465049

[B40] Aban JL Ramajo V Arellano JL Oleaga A Hillyer GV Muro A A fatty acid binding protein from Fasciola hepatica induced protection in C57/BL mice from challenge infection with Schistosoma bovis Vet Parasitol 1999 83 107 121 10.1016/S0304-4017(99)00053-9 10392967

[B41] Liu S Dong W Kong T Preparation and characterization of immunoglobulin yolk against the venom of Naja naja atra Indian J Exp Biol 2010 48 778 785 21341535

[B42] Ruan GP Ma L He XW Meng MJ Zhu Y Zhou MQ Hu ZM Wang XN Efficient production, purification, and application of egg yolk antibodies against human HLA-A*0201 heavy chain and light chain (beta2m) Protein Expr Purif 2005 44 45 51 10.1016/j.pep.2005.03.013 16199353

[B43] Ko KY Ahn DU Preparation of immunoglobulin Y from egg yolk using ammonium sulfate precipitation and ion exchange chromatography Poult Sci 2007 86 400 407 17234857 10.1093/ps/86.2.400

[B44] de Moraes MH Guarneri AA Girardi FP Rodrigues JB Eger I Tyler KM Steindel M Grisard EC Different serological cross-reactivity of Trypanosoma rangeli forms in Trypanosoma cruzi-infected patients sera Parasit Vectors 2008 1 20 10.1186/1756-3305-1-20 18611261 PMC2475519

[B45] Johnston KL Wu B Guimaraes A Ford L Slatko BE Taylor MJ Lipoprotein biosynthesis as a target for anti-Wolbachia treatment of filarial nematodes Parasit Vectors 2010 3 99 10.1186/1756-3305-3-99 20946650 PMC2964653

[B46] Liu F Lu J Hu W Wang SY Cui SJ Chi M Yan Q Wang XR Song HD Xu XN Wang JJ Zhang XL Zhang X Wang ZQ Xue CL Brindley PJ McManus DP Yang PY Feng Z Chen Z Han ZG New perspectives on host-parasite interplay by comparative transcriptomic and proteomic analyses of Schistosoma japonicum PLoS Pathog 2006 2 e29 10.1371/journal.ppat.0020029 16617374 PMC1435792

[B47] Liu F Hu W Cui SJ Chi M Fang CY Wang ZQ Yang PY Han ZG Insight into the host-parasite interplay by proteomic study of host proteins copurified with the human parasite, Schistosoma japonicum Proteomics 2007 7 450 462 10.1002/pmic.200600465 17211827

[B48] Van Dam GJ Bergwerff AA Thomas-Oates JE Rotmans JP Kamerling JP Vliegenthart JF Deelder AM The immunologically reactive O-linked polysaccharide chains derived from circulating cathodic antigen isolated from the human blood fluke Schistosoma mansoni have Lewis x as repeating unit Eur J Biochem 1994 225 467 482 10.1111/j.1432-1033.1994.00467.x 7925469

[B49] Bergwerff AA van Dam GJ Rotmans JP Deelder AM Kamerling JP Vliegenthart JF The immunologically reactive part of immunopurified circulating anodic antigen from Schistosoma mansoni is a threonine-linked polysaccharide consisting of – > 6)-(beta-D-GlcpA-(1 – > 3))-beta-D-GalpNAc-(1 – > repeating units J Biol Chem 1994 269 31510 31517 7989318

[B50] Masciadri B Areces LB Carpinelli P Foiani M Draetta G Fiore F Characterization of the BUD31 gene of Saccharomyces cerevisiae Biochem Biophys Res Commun 2004 320 1342 1350 10.1016/j.bbrc.2004.05.228 15303280

[B51] Saha D Khandelia P O'Keefe RT Vijayraghavan U Saccharomyces cerevisiae NineTeen complex (NTC)-associated factor Bud31/Ycr063w assembles on precatalytic spliceosomes and improves first and second step pre-mRNA splicing efficiency J Biol Chem 2012 287 5390 5399 10.1074/jbc.M111.298547 22215661 PMC3285318

[B52] Dunne DW Jones FM Doenhoff MJ The purification, characterization, serological activity and hepatotoxic properties of two cationic glycoproteins (alpha 1 and omega 1) from Schistosoma mansoni eggs Parasitology 1991 103 Pt 2 225 236 1745548 10.1017/s0031182000059503

[B53] Fitzsimmons CM Schramm G Jones FM Chalmers IW Hoffmann KF Grevelding CG Wuhrer M Hokke CH Haas H Doenhoff MJ Dunne DW Molecular characterization of omega-1: a hepatotoxic ribonuclease from Schistosoma mansoni eggs Mol Biochem Parasitol 2005 144 123 127 10.1016/j.molbiopara.2005.08.003 16143411

[B54] Everts B Perona-Wright G Smits HH Hokke CH van der Ham AJ Fitzsimmons CM Doenhoff MJ van der Bosch J Mohrs K Haas H Mohrs M Yazdanbakhsh M Schramm G Omega-1, a glycoprotein secreted by Schistosoma mansoni eggs, drives Th2 responses J Exp Med 2009 206 1673 1680 10.1084/jem.20082460 19635864 PMC2722183

[B55] Steinfelder S Andersen JF Cannons JL Feng CG Joshi M Dwyer D Caspar P Schwartzberg PL Sher A Jankovic D The major component in schistosome eggs responsible for conditioning dendritic cells for Th2 polarization is a T2 ribonuclease (omega-1) J Exp Med 2009 206 1681 1690 10.1084/jem.20082462 19635859 PMC2722182

[B56] Chaudhuri J Martin RW Donahue MJ Tryptophan hydroxylase and aromatic L-amino acid decarboxylase activities in the tissues of adult Ascaris suum Int J Parasitol 1988 18 341 346 10.1016/0020-7519(88)90143-9 3397216

[B57] Cass CL Johnson JR Califf LL Xu T Hernandez HJ Stadecker MJ Yates JR Williams DL Proteomic analysis of Schistosoma mansoni egg secretions Mol Biochem Parasitol 2007 155 84 93 10.1016/j.molbiopara.2007.06.002 17644200 PMC2077830

[B58] Braschi S Borges WC Wilson RA Proteomic analysis of the schistosome tegument and its surface membranes Mem Inst Oswaldo Cruz 2006 101 Suppl 1 205 212 17308771 10.1590/s0074-02762006000900032

[B59] Mulvenna J Moertel L Jones MK Nawaratna S Lovas EM Gobert GN Colgrave M Jones A Loukas A McManus DP Exposed proteins of the Schistosoma japonicum tegument Int J Parasitol 2010 40 543 554 10.1016/j.ijpara.2009.10.002 19853607

[B60] Delcroix M Medzihradsky K Caffrey CR Fetter RD McKerrow JH Proteomic analysis of adult S. mansoni gut contents Mol Biochem Parasitol 2007 154 95 97 10.1016/j.molbiopara.2007.03.008 17451823 PMC2732360

