# Supplementary material for: Identification and profiling of circulating antigens by screening with the sera from schistosomiasis japonica patients
Source: Parasit Vectors. 2012 Jun 11;5:115. doi: 10.1186/1756-3305-5-115 (PMC3419666; doi:10.1186/1756-3305-5-115)
Supplement: Additional file 1 — Table S1. The detailed information of schistosomiasis patients. [file 1756-3305-5-115-S1.doc]

| ID | Gender | Age | EPG | Occupation | Education | History of schistosome infection | Other helminthes infection |
| --- | --- | --- | --- | --- | --- | --- | --- |
| 1 | male | 56 | 24 | fisherman | primary school | No | No |
| 2 | female | 50 | 283 | fisherman | primary school | No | No |
| 3 | male | 49 | 13 | farmer | primary school | Yes | No |
| 4 | male | 43 | 19 | farmer | middle school | Yes | Yes |
| 5 | female | 42 | 19 | farmer | primary school | Yes | No |
| 6 | male | 57 | 21 | farmer | middle school | Yes | Yes |
| 7 | male | 42 | 11 | farmer | primary school | Yes | Yes |
| 8 | male | 46 | 32 | farmer | middle school | Yes | Yes |
| 9 | male | 12 | 37 | student | primary school | No | Yes |
| 10 | male | 59 | 35 | farmer | primary school | No | No |

**Table S1 The detailed information of schistosomiasis patients**
