# Supplementary material for: Identification and profiling of circulating antigens by screening with the sera from schistosomiasis japonica patients
Source: Parasit Vectors. 2012 Jun 11;5:115. doi: 10.1186/1756-3305-5-115 (PMC3419666; doi:10.1186/1756-3305-5-115)
Supplement: Additional file 2 — Table S2. The peptide sequences of the associated proteins. [file 1756-3305-5-115-S2.doc]

**Table S2** The peptide sequences of the associated proteins

| **Accession no.** | **Annotation** | **Score** | **Start** | **End** | **Sequence** |
| --- | --- | --- | --- | --- | --- |
| CAX70049.1 | Protein BUD31 homolog | 6.8356 | 83 | 91 | (K)IADANLIAK(W) |
|  |  | 5.5225 | 69 | 82 | (K)AISKELYEFCIKEK(I) |
|  |  | 4.6469 | 7 | 27 | (R)NKKPPPEGWELIEPTIEELNR(K) |
| CAX70531.1 | ribonuclease | 5.7385 | 32 | 91 | (R)VNAVAFSEDIPLNETAWASSGYAPLHVEEAYVMVSNNCFIAAGIYVVLLIFSGVQYYFNK(R) |
| AAX28592.2 | SJCHGC06971 protein | 6.3786 | 84 | 96 | (K)HSESGGSEVGSDR(V) |
|  |  | 5.5267 | 52 | 83 | (K)SSPDYDNASGSSSLIHASDESFHQIA KAIPSK(H) |
|  |  | 5.466 | 1 | 16 | (-)MTSSPSVNIPSSHHHR(H) |
|  |  | 4.7753 | 129 | 137 | (R)NGSSAQCSR(L) |
|  |  | 4.6129 | 109 | 118 | (R)CVETIIVHIR(N) |
| AAX28446.2 | SJCHGC04754 protein | 6.0598 | 49 | 62 | (K)EPETWSIIFHDIEK(Y) |
